# Avulsion Fracture of Brachioradialis Muscle Origin: An Exceedingly Rare Entity: A Case Report

**DOI:** 10.5704/MOJ.1607.010

**Published:** 2016-07

**Authors:** G Behera, G Balaji, J Menon, D Sharma, VK Komuravalli

**Affiliations:** Jawaharlal Institute of Postgraduate Medical Education and Research (JIPMER), Puducherry, India

**Keywords:** Brachioradialis muscle, avulsion injury, non-operativetreatment

## Abstract

Avulsion fracture of the brachioradialis origin at its proximal attachment on the lateral supracondylar ridge of the distal humerus is exceedingly rare, and only two cases have been reported in the literature so far. In this article, we present a 38 years old patient who sustained a closed avulsion fracture of the lateral supracondylar ridge of left humerus at the proximal attachment of brachioradialis following a fall backwards on outstretched hand after being struck by a lorry from behind while riding on a two-wheeler (motorcycle). He was managed with above elbow plaster for four weeks followed by elbow and wrist mobilization. At final followup, the patient had painless full range elbow motion with good elbow flexion strength. The unique mechanism by which this avulasion fracture occurred is explained on the basis of the mode of injury, position of the limb and structure and function of the brachioradialis muscle.

## Introduction

Avulsion fracture of brachioradialis origin is an exceedingly rare entity. Thus far only two cases have been reported in the English literature^1-2^. In this report, we discuss in detail the mechanism of injury of this type of avulsion fracture, the importance of proper imaging and the line of management, with brief review of the literature pertinent to this rare injury.

## Case Presentation

A 38 years old male patient presented to the emergency room with left elbow pain following a road traffic injury. He was travelling in a motorcycle when he was hit by a lorry from the rear and fell backward on the outstretched hand. On physical examination, there was minimal swelling around the elbow but significant tenderness with crepitus on the lateral supracondylar ridge just proximal to the lateral epicondyle. He had restriction of active terminal elbow extension by 10 degrees with near normal active elbow flexion, pronation and supination. Active flexion and extension at wrist were painful along with the painful terminal elbow extension. There was significant pain at the lateral distal humerus when active elbow flexion against resistance was performed in the mid-pronated position of the forearm. There was no distal neurovascular deficit.

**Fig. 1:**
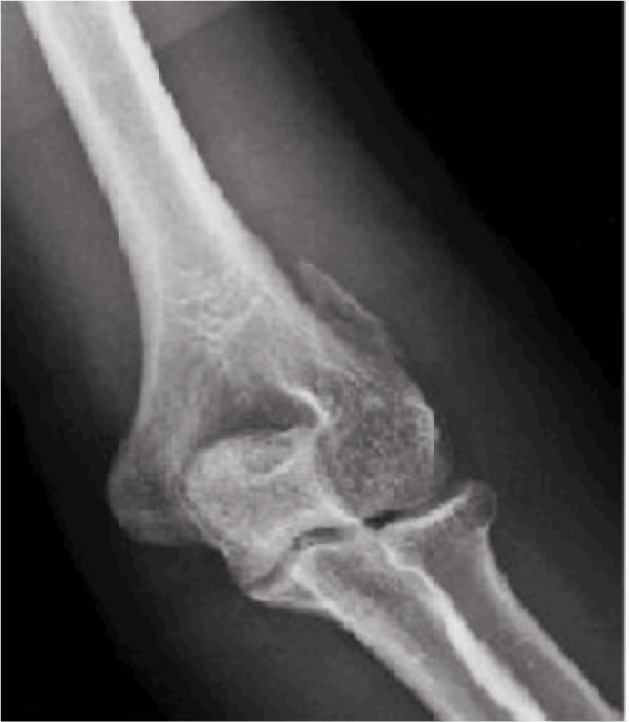
AP radiograph of left elbow avulsion fracture of brachioradialis origin.

**Fig. 2:**
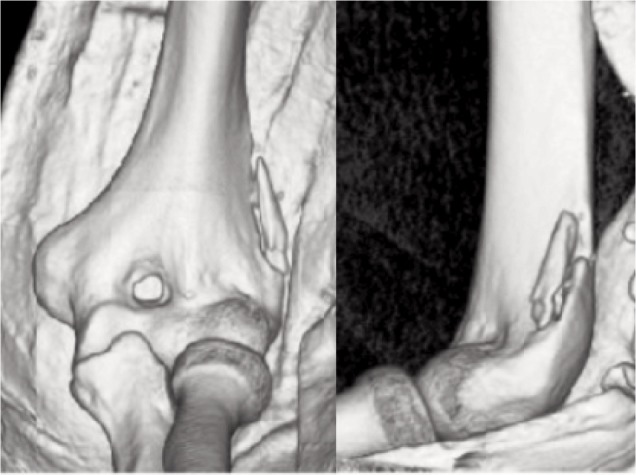
3D Reconstruction of left distal humerus demonstrating avulsion fracture of brachioradialis muscle origin at the proximal part of lateral supracondylar ridge.

Antero-posterior (AP) plain radiograph of the left elbow showed a fracture of the lateral distal humerus at the proximal part of the lateral supracondylar ridge above the epicondyle (Figure 1). Computed tomography (CT) along with 3-dimensional reconstructions (3D) of the elbow further demonstrated a 3.0 cm crescentic fragment avulsed from the lateral supracondylar ridge of the distal humerus 1.2cm above the lateral epicondyle with 3mm anterolateral displacement (Figure 2). Based on the mechanism of the injury and the particular location and nature of the fracture fragment, a diagnosis of avulsion fracture of the left brachioradialis muscle from its origin at the left distal humerus attachment was made.

**Fig. 3:**
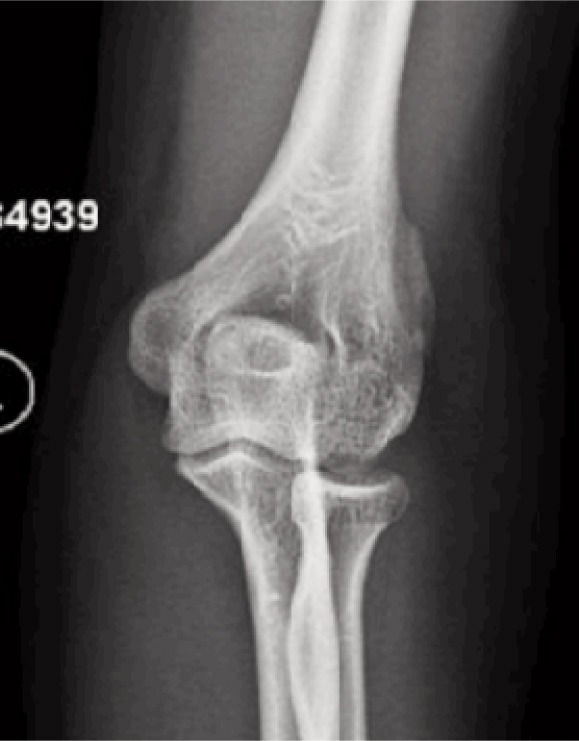
Follow up radiograph at 14 weeks shows good callus and bony union.

In view of the minimally displaced fracture, the patient was managed non-operatively with a long arm plaster cast with the elbow in 90 degree flexion and wrist held in midpronated position to avoid stresses on the wrist extensors and the brachioradialis muscle. At the end of four weeks, the cast was removed, and clinical reassessment revealed mild tenderness at the fracture site with minimal pain upon active resisted elbow flexion in the semi-pronated position of the forearm. He was started on rehabilitation with gradual elbow, wrist and finger range of motion exercises and was instructed to abstain from lifting heavy objects for a period of 12 weeks. By 14 weeks, there was no local tenderness and the resisted active elbow flexion test was painless with full strength across wrist and elbow. Follow-up radiograph showed bone healing with good callus formation (Figure 3).

## Discussion

An avulsion fracture, usually seen in athletes, occurs when a small chip of bone attached to a ligament or tendon is pulled off from the main mass of the bone due to external force or forceful eccentric contraction of muscle. The common sites of humeral avulsion fractures include the fractures of lateral or medial epicondyles and fractures of greater or lesser tuberosities^3^. Avulsion fracture of the brachioradialis muscle origin at the lateral distal humerus is extremely rare, and only two cases have been reported till now^1-2^.

Brachioradialis is the most superficial muscle along the radial side of the forearm and forms the lateral border of the cubital fossa. It arises from the proximal two-thirds of the lateral supracondylar ridge of the humerus and the anterior surface of the lateral intermuscular septum and inserts on the styloid process of the distal radius^4^. Brachioradialis along with the extensor carpi radialis longus and brevis form the dorsal mobile wad of the forearm and are innervated by the radial nerve. Given the location of its proximal and distal attachments, this muscle has a significant mechanical advantage for elbow flexion, specifically in the mid-pronated forearm^5^.

Guettler and Mayo^1^ reported one case of type I open (Gustillo-Anderson) distal humerus fracture secondary to avulsion of the brachioradialis muscle origin in a man who was thrown backwards after being struck by a fireworks shell. According to them, when the victim was thrown, he attempted to prevent the fall with his arm placed backward, forearm pronated, elbow and shoulder extended which resulted in eccentric contraction of the muscle causing the avulsion fracture with a bony spike and a grade I open fracture. They managed him with debridement without any fixation followed by 48 hours of antibiotics with good bony union by six months.

Marchant *et al*^2^ reported a similar type of case in a professional lacrosse player who sustained injury following a direct check on the lateral distal humerus by a defending player’s stick. Their patient had an associated superficial radial nerve injury along with avulsion fracture of the brachioradialis origin. The patient was treated conservatively with elbow splints with complete recovery of superficial radial nerve by eight weeks.

The mechanism of injury in our case is very similar to that reported by Guettler and Mayo^1^ as here the patient fell backward on his outstretched hand after being struck by a lorry from the rear when he was travelling on a motorcycle. Our patient while being thrown would have attempted to prevent the fall with his arm placed backward, forearm pronated, elbow and shoulder extended. This position of the upper limb led to the forceful eccentric contraction of the brachioradialis muscle which resulted in the avulsion fracture of the muscle from its origin at the lateral supracondylar ridge of the distal humerus. This is the first case reported following road traffic injury.

On imaging, we found a 3cm crescentic bony fragment avulsed from the lateral distal humerus 1.2cm above the epicondyle with anterolateral displacement. The typical location over proximal part of the lateral supracondylar ridge, crescentic shape and anterolateral displacement of the fracture fragment in our patient were consistent with the avulsion fracture of brachioradialis muscle from its origin.

One of the cases reported as in our case had been treated by immobilization with splints while the other reported case which had debridement for the open wound. The rationale for treating this avulsion fractures non-operatively is that undisplaced or minimally displaced avulsion fracture of muscle usually heal with minimal sequelae unless the bony fragment is a part of joint congruency and the muscle served as an important stabilizer for the joint. Also, open surgery carries a very high risk of injury to the radial nerve in view of its close proximity to the avulsion fracture fragment apart from the implant related complications and the need for second surgery to remove the implants.

Avulsion fracture of brachioradialis muscle origin is exceedingly rare. Proper history, knowledge of the exact mechanism of injury, meticulous clinical examination and appropriate imaging will help in the diagnosis of such a rare injury. Non-operative treatment with splints for four weeks followed by mobilization is adequate for good fracture healing and functional outcome.

## References

[1] Guettler JH, Mayo DB (2001). Avulsion fracture of the origin of the brachioradialis muscle. Am J Orthop.

[2] Milford H, Marchant Ralph A, Gambardella Luga Podesta (2009). “Superficial radial nerve injury after avulsion fracture of the brachioradialis muscle origin in a professional lacrosse player: A case report. J Shoulder Elbow Surg.

[3] Kobayashi Y, Oka Y, Ikeda M, Munesada S (2000). Avulsion fracture of the medial and lateral epicondyles of the humerus. J Shoulder Elbow Surg.

[4] Susan Standring (2008). Gray’s anatomy: The anatomical basis of clinical practice.

[5] An KN, Morrey BF, Morrey BF (1985). Biomechanics of the elbow. The elbow and its disorders.

